# 1311. Mycoplasma and Ureaplasma Septic Arthritis - A Diagnostic Challenge

**DOI:** 10.1093/ofid/ofad500.1150

**Published:** 2023-11-27

**Authors:** Lalithaa Thirunavukarasu Murugan, Eric Cober, Lucileia Johnson

**Affiliations:** Cleveland Clinic, Cleveland, Ohio; Cleveland Clinic Foundation, Cleveland, OH; Cleveland Clinic, Cleveland, Ohio

## Abstract

**Background:**

Mycoplasma and ureaplasma septic arthritis (MUSA) is difficult to diagnose as these usually require special media or polymerase chain reaction (PCR) to identify. Empiric antibiotics for septic arthritis do not cover these organisms, causing treatment delays. We aimed to study the characteristics and outcomes of patients with MUSA.

**Methods:**

This is a retrospective review of patients (pts) with MUSA admitted to Cleveland Clinic from January 2005 to June 2022. We included pts, 18 years or older, with culture or PCR positive for mycoplasma or ureaplasma from joint tissue or synovial fluid (SF). 7 pts met inclusion criteria.

**Results:**

Median age at diagnosis was 29 years (range 23 - 59). 4/7 pts were male. All had one or more underlying immunocompromising condition and received immunosuppressants within a year prior to diagnosis, 4/7 received rituximab. Immunoglobulin G levels were checked in 5/7 pts within a month around diagnosis, and 3/5 had a level below 500 mg/dl (reference range 717 - 1411).

It took a median of 54 days (range 3 - 402) from initial encounter concerning septic arthritis to diagnosis of MUSA. 5/7 had monoarticular MUSA. 4/7 had native joint arthritis and 3 had periprosthetic joint infection.

SF study closest to diagnosis had a median total nucleated cells of 126,000/µL (range 59 – 204,498), with median neutrophil of 86% (range 62 - 98). Diagnosis was made by culture and PCR in 5 and 2 pts, respectively. Median C- reactive protein around diagnosis was 10.5 mg/dl (range 1.5 - 29.7).

Of the 7 pts, 6 required surgical intervention, with 3 requiring multiple surgeries. Gross purulence was a hallmark intra-operative finding in all patients. 1 out of 7 pts was on indefinite doxycycline suppression. Another one died of advanced AIDS after 2 days of MUSA treatment. Remaining 5/7 received antibiotics for a median of 108 days (range 61 – 1027), of which 3 received dual antibiotics. Excluding the patient who died of AIDS, the remaining recovered completely without relapse during a minimum 4-year follow up.

**Figure:**
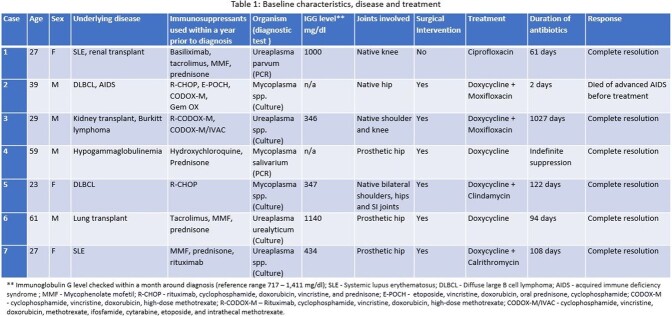

**Conclusion:**

To our knowledge, this is the largest case series of MUSA. All affected patients were immunocompromised, highlighting the need for high index of clinical suspicion in this population to avoid diagnostic delays. Although challenging to diagnose, it appears that MUSA can be effectively treated.

**Disclosures:**

**All Authors**: No reported disclosures

